# Supplementary material for: Lasting lockdown love? Problem behaviour and pandemic and non-pandemic related risk factors influencing the owner-dog relationship in a UK cohort of dogs reaching early adulthood
Source: PLoS One. 2025 Feb 12;20(2):e0316166. doi: 10.1371/journal.pone.0316166 (PMC11819559; doi:10.1371/journal.pone.0316166)
Supplement: S1 Appendix — (PDF) [file pone.0316166.s001.pdf]

S1 Appendix: 21-Month Survey Questions Relevant to Study

CONSENT QUESTIONS AND INCLUSION CRITERIA:

Pandemic Puppies Follow-Up Survey, 21 Months

In November-December 2020, you were one of 7545 owners who kindly responded to a survey run by our team at the Royal Veterinary College (RVC) – thank you! You indicated that you would be willing to be involved in further research about your dog and have therefore been specifically chosen to take part in our next exciting research project following the progress of puppies bought during the 2020 phase of the COVID-19 pandemic.

If you no longer have the dog for which you completed the survey, we would still like to hear from you. You will be able to indicate this at the very start of the study and will then be taken to a modified version of the survey which will take no more than 5 minutes to complete.

If you still own your dog but you do not wish to hear from us anymore, please fill in the very start of the survey to indicate this, following this you will not receive any further contact from us.

What is this further research about?

This research, generously funded by Battersea Dogs and Cats Home, aims to better understand and support the needs of owners such as yourself, who are raising dogs during this unprecedented period of our lives. By taking part, your unique views will enable us to tailor advice to owners to help support an entire generation of dogs.

What does the study involve?

We are inviting you to take part in a series of three short surveys over the next year (when your dog reaches 21, 24 and 27 months), which will explore the long-term impact of buying a puppy, and your puppy growing up, during the COVID-19 pandemic. This will include exploring impacts upon your dog, but also yourself as their owner.

To take part, you simply need to answer a series of questions online which will take approximately 25-30 minutes (or under 5 minutes if you are opting out/no longer have the dog you previously completed the survey for). The link you have been provided with in the accompanying email is unique to you and your dog and should not be forwarded to others. You will have 28 days in total from receiving the first email to complete the survey, after which time your unique link will expire.

You have received this survey as it is now 21 months since the date of birth you provided for your dog in the 2020 survey. After completing this survey, if you consent to do so, we will send further surveys 24 months and 27 months after your dog's date of birth, so you can update us on their behaviour, health, and your growing relationship with them.

We ask that you answer the survey for the dog you answered the initial survey for in November-December 2020. As a reminder, if you bought more than one puppy between 23 March 2020 and 31 December 2020, you were asked to answer for the youngest puppy. If two puppies were the same age (e.g., littermates) then you were asked to answer for the puppy whose name came first alphabetically.

The survey is ideally to be completed by the person who was/is most involved in the care of this dog. If possible, this would be the same person who completed the initial survey last autumn/winter and provided us with this email address.

How do I complete the survey?

Once you have started the survey, you will be able to save and answer the questions in more than one session using your device of choice (e.g., smartphone, PC) until you submit the survey to us. After this you will no longer be able to access your responses. To pause the survey and return later simply press the 'Save and Return Later' button and you will be provided with an auto-generated return code, which you will be required to enter upon returning to the survey in order to continue. If you accidently press your browsers 'back' button whilst completing the questions and lose the survey, it can be reloaded by refreshing the page/pressing F5.

What will happen to my information?

Your information will be kept by the RVC in accordance with GDPR. Only the scientists undertaking this study (Dr. Rowena Packer, Dr. Dan O'Neill, Dr. Zoe Belshaw, Dr. Claire Brand, Dr. Camilla Pegram, Dr Fiona Dale, Dr. Kim Stevens and student members of their team) will have access to your data. This data will be anonymised and used for research and only anonymised results and data will be published. Publications may include quotes from your written responses, but these will be anonymised, and you will not be identifiable from any quotes. This study has received ethical approval from the RVC Social Science Research Ethical Review Board (SR2020-0259). If you have any further questions about the study, please contact Dr. Rowena Packer and Dr. Claire Brand (pandemicpuppies@rvc.ac.uk).

Opting out

You can opt out of this survey or the 24 or 27 month surveys when they are released, which means any data you have already submitted to us remains with us, but we do not contact you regarding further involvement in the study, e.g., at future time points of this survey, and do not send you any further reminders for this survey time point. There is an option to do this at the very beginning of this survey, in addition to the option to be removed from our mailing list which we use to update participants about project news and publications.

Can I change my mind and withdraw my data?

Should you wish to withdraw your data from the study at any point, please contact Dr. Rowena Packer and Dr. Claire Brand (pandemicpuppies@rvc.ac.uk). Any data that you request to be withdrawn will be permanently deleted.

What if taking part raises concerns?

If this study raises any concerns about your dog's welfare, then please contact a veterinary surgeon to discuss. Resources on specific canine welfare issues raised will also be included at the end of the survey. If this study raises any concerns about your own mental health, then please access these NHS resources: www.nhs.uk/conditions/stress-anxiety-depression/mental-health-helplines/

Q1
I confirm that:
1. I completed the first 'Pandemic Puppies' survey in 2020, having brought a puppy of any breed or crossbreed home aged under 16 weeks between 23 March 2020 and 31 December 2020.
2. I am over 18 years of age.
3. I am a resident of the UK.
4. I have read and understood the above information and give consent for my answers to be used for this research study and any resulting publications.

Q2
Do you still own the dog you told us about in the 2020 'Pandemic Puppies' survey (that you purchased between 23rd March-31st December 2020)?
N.B. As a reminder, if you purchased more than one puppy in the time period above, we asked you to answer for the youngest dog, or in the case of littermates, the dog whose name came first alphabetically.
1. Yes – I still have my dog **[SURVEY LOGIC – skip questions 3-14]**
2. No – I have rehomed/sold/given away this dog [**SURVEY LOGIC – skip questions 7-13]**
3. No – my dog has passed away/been put to sleep [**SURVEY LOGIC – skip questions 3-6 and 12-13]**
4. No – my dog was stolen/lost and is still missing **[SURVEY LOGIC – skip questions 3-11]**

Q3
If you are comfortable doing so, please describe in your own words why you decided to rehome, sell or give away or otherwise stop owning this dog. **[free text]**

Q4
Which month/year did you rehome, sell or give away this dog?
Drop down month list
Drop down year list (2020-2022)

Q7
We're sorry to hear that your dog is no longer with you. If you feel comfortable doing so, we'd like to ask you to answer a few questions about what happened to enable us to help other owners. Firstly, how did your dog die?
1. Put to sleep (euthanised) **[SURVEY LOGIC – skip question 9]**
2. Natural death (not put to sleep) **[SURVEY LOGIC – skip question 8]**
3. Accidental death (e.g., run over) **[SURVEY LOGIC – skip question 8]**

Q8
If you are comfortable doing so, please describe in your own words what led to the decision for your dog to be put to sleep. **[free text]**

Q10
Please indicate which month/year your dog was put to sleep or passed away.
Drop down month list
Drop down year list (2020-2022)

Thank you for giving your time to explain why you no longer have your dog.
Please indicate below whether you would still be interested in receiving project updates.

Q14

Would you like to receive project updates from the RVC about the Pandemic Puppies study?

1. Yes, I would like to receive updates about the research you are carrying out (please do not delete my contact details) **[SURVEY LOGIC – send to the end of the survey, debrief section 2]**
2. No, I would rather not to hear from you again in the future (please delete my contact details) **[SURVEY LOGIC – send to the end of the survey, debrief section 2]**

Q15

Are you willing to take part in additional surveys about your experiences with your dog?

This would greatly help our overall understanding of dog welfare and enable us to help improve the lives of dogs in the future.

N.B. Surveys will take ~25 minutes, with the first survey to be completed within 28 days of the initial survey link being sent via email. Links to the following two surveys will be sent in 3 months and 6 months, respectively.

1. Yes, I would like to take part in further research about my dog
2. No, but I would like to receive updates about the research you are carrying out (please do not delete my contact details) **[SURVEY LOGIC – send to the end of the survey, debrief section 1]**
3. No, I would rather not take part in further research about my dog and would prefer not to hear from you again in the future (please delete my contact details) **[SURVEY LOGIC – send to the end of the survey, debrief section 1]**

DOG HEALTH QUESTIONS (PARTICIPANTS WHO STILL OWN THEIR DOG):

In this section we would like to understand any changes to your dog's access to veterinary care since the last survey (in November/December 2020/). In addition, we would like to understand any changes surrounding your dog's health since the last survey for which you may or may not have sought veterinary advice.

Some of these questions are very similar to those asked in the first survey in November/December 2020, but for some owners the answers may have changed.

Taking the time to answer these questions will greatly help our overall understanding of dog welfare and veterinary care in the UK and enable us to help improve the lives of dogs in the future.

Q16

Is your dog currently registered with a veterinary clinic(s)?

1. Yes [SURVEY LOGIC – skip question 17]
2. No [SURVEY LOGIC – skip questions 18-25]

Q20

Does your dog have any ongoing health problem(s) that still require appointments/treatment/monitoring?

N.B. This does NOT include routine or preventative care (e.g., neutering, vaccination, microchipping, nail clipping, anal gland expression, routine check-ups, worming treatments, etc.).

1. No
2. I'm not sure
3. Yes (please describe below) [**free text**]

Q32

Has your dog been neutered (spayed/castrated), and if so, at what age?

1. Yes, aged under 6 months **[SURVEY LOGIC – skip questions 33 and 34]**
2. Yes, aged over 6 months, and I did not breed from them first **[SURVEY LOGIC – skip questions 33 and 34]**
3. Yes, aged over 6 months, and I bred from them first **[SURVEY LOGIC – skip question 33]**
4. No, but I intend to have them neutered when they are older
5. No, not yet, I haven't decided whether to neuter them

DOG BEHAVIOUR AND LIFESTYLE QUESTIONS (PARTICIPANTS WHO STILL OWN THEIR DOG):

In this section we would like to understand any changes surrounding your dog's behaviour and lifestyle since the last survey (in November/December 2020).
Once again, we understand that some of these questions are very similar to those asked in the first survey, but for some owners the answers may have changed.

Taking the time to answer these questions will greatly help our overall understanding of dog welfare and enable us to help improve the lives of dogs in the future.

Q35

Since the last survey (in November/December 2020), have you or someone in your household attended any training classes with your dog?
Please select **all options** that apply.

1. Yes, in-person puppy classes (whilst my puppy was under the age of 16 weeks)
2. Yes, in-person classes for adult dogs (over the age of 16 weeks, such as obedience, gundog training, scentwork, ringcraft, agility classes, etc.)
3. Yes, online classes (whilst my puppy was under the age of 16 weeks)
4. Yes, online classes for adult dogs (over the age of 16 weeks, such as obedience, gundog training, scentwork, ringcraft, agility classes, etc.)
5. No, I/we attended in person puppy classes whilst my dog was under the age of 16 weeks but haven't attended any other classes since completing the last survey
6. No, not as yet but I plan to in the future
7. No, my dog's circumstances have prevented us attending (e.g., my dog was ill, my dog was injured, etc.)
8. No, my personal circumstances have prevented us attending (e.g., I was ill, the classes were at inconvenient times, classes were too expensive, etc.)
9. No, I chose not to because I am a professional dog trainer/behaviourist and have conducted equivalent training myself
10. No, I wanted to, but there weren't any classes running
11. No, I wanted to, but the classes were fully booked
12. No, and I do not intend to

Q36

Have you or anyone in your household ever used any of the following aids or methods on/with your dog, to try and change any aspect of their behaviour?

Please select **all options** that apply.

[Columns]:

a. Yes
b. No

[Rows]:

1. Allowing sniffing time/interaction with the environment
2. Anti-bark 'Husher' muzzle
3. Bark-activated citronella/vibration/ultrasonic collar
4. Choke/check chain
5. Clicker training
6. Electric collar
7. Electronic boundary fence
8. Food/treats
9. Harness
10. Head collar
11. Lead corrections (e.g., quickly yanking back on/jerking the lead if your dog pulls)
12. Pet Corrector™
13. Physical correction (e.g., smacking, tapping their nose, hitting, pinching)
14. Physical touch, e.g., stroking or patting
15. Physically moving your dog (e.g., pushing on your dog's hindquarters to get them into a sit, pushing them off furniture or if they jump up at you)
16. Playing with other dogs
17. Playing with you/another household member
18. Prong collar
19. Rattle bottle/cans/discs as a distractor
20. Rubbing their nose in faeces/urine if they toilet in an inappropriate location
21. Shouting at them or telling them off
22. "Time out": shutting your dog away within the home, away from people (e.g., in another room, in their crate)
23. "Time out": shutting your dog outdoors (e.g., in the garden/yard or an outside kennel)
24. Slip lead
25. Toys
26. Verbal praise
27. Water pistol/spray bottle

Q37

At any point in time have you or anyone in your household used any other aids or methods not listed previously on/with your dog to try and change any aspect of their behaviour?

1. No
2. Yes (please describe below) **[free text]**

Q39(a)
At any point in time since the last survey (in November/December 2020) have you considered that your dog has a problem with the following behaviours? If so, have you sought advice either formally or informally?
Please select **all options** that apply.
[Columns]:

a. No
b. Yes, but I did not seek advice
c. Yes, advice from a veterinary professional
d. Yes, advice from a dog trainer
e. Yes, advice from a dog behaviourist
f. Yes, advice from my dog's breeder
g. Yes, advice from another source (e.g., friend, family book, social media, etc.)
h. I'm not sure

[Rows]:

1. Pulling on their lead
2. Jumping up at people
3. Barking at other dogs
4. Not coming back when called
5. Fear of loud sounds (e.g., fireworks, thunderstorms)
6. Chasing (e.g., cats, wildlife, traffic)
7. Clinginess (e.g., following you, sitting close)
8. Anxiety/fear around other dogs
9. Anxiety/fear around people in your household (including you)
10. Anxiety/fear around unfamiliar people
11. Aggression towards other dogs
12. Aggression towards people in your household (including you)
13. Aggression towards unfamiliar people
14. Guarding of food, toys, or other items
15. Mouthing
16. Barking or howling
17. Being destructive
18. Toileting (weeing or pooing) indoors
19. Eating their or other dogs' faeces
20. Mounting/humping other dogs, people or objects
21. Other (please describe below) **[Q39(b)]**

Q39(b)
Please describe here in your own words, any other behaviours not listed above that your dog has a problem with, including where you sought advice if relevant. **[free text]**

Q40

Has your dog ever performed any of the following behaviours?

Please select **all options** that apply.

[Columns]:

a. Yes
b. No
c. I'm not sure

[Rows]:

1. Repeatedly spins in a circle and/or chases their tail, i.e., turns in a circle more than three times, and may or may not catch their tail **[SURVEY LOGIC – send to question 41 if option (a) 'Yes' was selected]**
2. Chases, stalks, pounces, stares or bites at lights, reflections and/or shadows **[SURVEY LOGIC – send to question 42 if option (a) 'Yes' was selected]**
3. Stares into the sky and snaps/bites (WITHOUT flies/insects being there), which may be accompanied by jumping, licking, and swallowing **[SURVEY LOGIC – send to question 43 if option (a) 'Yes' was selected]**
4. Repeatedly chews or sucks themselves (e.g., their feet, legs, side) or excessively licks or sucks other objects (e.g., surfaces, blankets) for more than 30 seconds at a time **[SURVEY LOGIC – send to question 44 if option (a) 'Yes' was selected]**
5. Repeatedly seeks out and eats non-food items (e.g., cloth, plastic, stones, faeces) **[SURVEY LOGIC – send to question 45 if option (a) 'Yes' was selected]**

Q48

What length of time is your dog is currently left at home alone (without any people present in the home) on a typical weekday?

N.B. This should be the longest period of time that your dog is left without a break/visit, e.g., if you leave your home between 8 am to 5 pm but either yourself or someone else visits your dog at 1 pm, the longest stretch they are left alone is 5 hours.

1. Never left alone
2. Under 1 hour
3. Up to 2 hours
4. Up to 3 hours
5. Up to 4 hours
6. Up to 5 hours
7. Up to 6 hours
8. Up to 7 hours
9. Up to 8 hours
10. Up to 9 hours
11. Up to 10 hours
12. Over 10 hours

Q50(a)
Since the last survey (in November/December 2020) has your dog shown any of the following behaviours when at home with you/other household members while you are relaxing (e.g., watching TV)?
Please select **all options** that apply.
[Columns]:

a. Yes
b. No

[Rows]:

1. Destructive behaviour (e.g., chewing and causing damage to household items such as furniture, not including their own toys/treats)
2. Scratches doors or around doors
3. Barks
4. Howls
5. Whines/cries
6. Wees in an inappropriate place, e.g., inside the home if usually housetrained
7. Poos in an inappropriate place, e.g., inside the home if usually housetrained
8. Spins (repeatedly turns in a circle)
9. Paces (walks repetitively around the home/room, unable to settle)

Q50(b)
Please describe here in your own words, any other behaviours not listed above that your dog has shown since the last survey (in November/December 2020) when at home with you/other household members while you are relaxing (e.g., watching TV)? **[free text]**

Q51

To your knowledge (e.g., from seeing evidence on your return home, on a video camera, being told by neighbours, etc.), since the last survey (in November/December 2020) has your dog shown any of the following behaviours when left at home alone (without any people present in the home)?

Please select **all options** that apply.

[Columns]:

a. Yes
b. I suspect they might
c. No
d. I don't know

[Rows]:

1. Destructive behaviour (e.g., chewing and causing damage to household items such as furniture, not including their own toys/treats) **[SURVEY LOGIC – send to question 53 if option (a) 'Yes' was selected]**
2. Scratches doors or around doors **[SURVEY LOGIC – send to question 53 if option (a) 'Yes' was selected]**
3. Barks **[SURVEY LOGIC – send to question 53 if option (a) 'Yes' was selected]**
4. Howls **[SURVEY LOGIC – send to question 53 if option (a) 'Yes' was selected]**
5. Whines/cries **[SURVEY LOGIC – send to question 53 if option (a) 'Yes' was selected]**
6. Wees in an inappropriate place, e.g., inside the home if usually housetrained **[SURVEY LOGIC – send to question 53 if option (a) 'Yes' was selected]**
7. Poos in an inappropriate place, e.g., inside the home if usually housetrained **[SURVEY LOGIC – send to question 53 if option (a) 'Yes' was selected]**
8. Spins (repeatedly turns in a circle) **[SURVEY LOGIC – send to question 53 if option (a) 'Yes' was selected]**
9. Paces (walks repetitively around the home/room, unable to settle) **[SURVEY LOGIC – send to question 53 if option (a) 'Yes' was selected]**

Q52

To your knowledge (e.g., from seeing evidence on your return home, on a video camera, being told by neighbours, etc.), since the last survey (in November/December 2020) has your dog shown any other behaviours not listed above when left at home alone (without any people present in the home)?

1. No **[SURVEY LOGIC – skip question 53]**
2. Yes (please describe below in your own words) **[free text]**

Q54

If you go out to work, do you take your dog to your place of work?

1. Not applicable, I don't go out to work
2. Yes, always
3. Yes, most times (at least half or more of my workdays)
4. Yes, occasionally (less than half of my workdays)
5. No, I don't want to
6. No, my workplace is not suitable for dogs
7. No, my workplace doesn't allow it
8. No, I have tried taking my dog to work previously but their behaviour was not suited to my work environment
9. No, I have not asked but may request to do this in the future
10. No, but another member of my household takes my dog to work with them
11. No, other reasons (please describe below) **[free text]**

LIVING WITH THEIR DOG QUESTIONS (PARTICIPANTS WHO STILL OWN THEIR DOG):

In this section we would like to understand how living with your dog has met, or not, any expectations you may have had before or soon after you purchased them.

Q63

Please consider the following questions and report approximately how often you and your dog take part in the activities (if ever).

How often do you…

[Columns]:

a. At least once a day
b. Once every few days
c. Once a week
d. Once a month
e. Never

[Rows]:

1. Play games with your dog?
2. Take your dog to visit people?
3. Give your dog food treats?
4. Kiss your dog?
5. Take your dog in the car?
6. Hug your dog?
7. Have your dog with you while relaxing, e.g., watching TV?
8. Groom your dog?

Q64

Please consider the following questions and report approximately how often (if ever) they apply.

How often…

[Columns]:

a. Once a day
b. Once a week
c. Once a month
d. Once a year
e. Never

[Rows]:

1. Do you tell your dog things you don't tell anyone else?
2. Do you feel that looking after your dog is a chore?
3. Does your dog stop you doing things you want to?
4. Do you feel that having a dog is more trouble than it is worth?

Q65

Please consider the following question and report approximately how often (if ever) it applies.

How often do you…

[Columns]:

a. Once a week
b. Once a fortnight
c. Once a month
d. A couple of times a year
e. Never

[Rows]:

1. Buy your dog presents?

Q66

Please consider the following statements and indicate your level of agreement with them, from strongly agreeing, through to strongly disagreeing.

[Columns]:

a. Strongly agree
b. Agree
c. Neither agree nor disagree
d. Disagree
e. Strongly disagree

[Rows]:

1. My dog helps me get through tough times
2. My dog is there whenever I need to be comforted
3. I would like to have my dog near me all the time
4. My dog provides me with constant companionship
5. If everyone else left me, my dog would still be there for me
6. My dog gives me a reason to get up in the morning
7. I wish my dog and I never had to be apart
8. My dog is constantly attentive to me
9. It is annoying that I sometimes have to change my plans because of my dog
10. It bothers me that my dog stops me doing things I enjoyed doing before I owned it
11. There are major aspects of owning a dog I don't like
12. My dog makes too much mess
13. My dog costs too much money

Q67

Please consider the following question and select the choice that best matches your answer.

[Columns]:

a. Very traumatic
b. Traumatic
c. Neither traumatic nor untraumatic
d. Untraumatic
e. Very untraumatic

[Rows]:

1. How traumatic do you think it will be for you when your dog dies?

Q68

Please consider the following question and select the choice that best matches your answer.

[Columns]:

a. Very hard
b. Hard
c. Neither hard nor easy
d. Easy
e. Very easy

[Rows]:

1. How hard it is to look after your dog?

COVID IMPACT QUESTIONS (PARTICIPANTS WHO STILL OWN THEIR DOG):

In this final section we would like to understand how the COVID-19 pandemic has impacted upon you/your household since you first brought your dog home in 2020 whilst under the age of 16 weeks.

Q72

Where do you currently work?

1. I work from home
2. I work at a location away from my home address
3. I work both from home and at a location away from my home address
4. N/A – I am unemployed
5. N/A – I am retired

Q73

Where do other adults in your household currently work?
Please select **all options** that apply.

1. N/A – I live alone/I am the only adult in my household
2. They work from home
3. They work at a location away from our home address
4. They work both from home and at a location away from our home address
5. N/A – they are unemployed
6. N/A – they are retired

Q78

Compared to when your dog was under 16 weeks, has there been a change in who is involved in your dog's care?

Please select **all options** that apply.

1. No, the person/people who care for my dog has stayed the same
2. Yes, my dog now also attends doggy daycare
3. Yes, my dog is now also walked by a dog walker
4. Yes, my dog is now also cared for by family member(s)
5. Yes, my dog is now also cared for by friend(s)
6. Yes, my dog is now also cared for by others via dog sharing services (e.g., BorrowMyDoggy)
7. Yes, other (please describe below) **[free text]**

Q79

Since you bought your dog home in 2020, have your household finances been affected by the COVID-19 pandemic?

1. My household finances are the same
2. My household finances are worse
3. My household finances are better
4. I'd rather not say
5. I'm not sure

Q81

Do you foresee any changes to your/your household's circumstances in the next three months that might make it easier or more difficult to continue owning your dog?

1. No **[SURVEY LOGIC – skip question 82]**
2. Yes – changes that might make it easier
3. Yes – changes that might make it more difficult

Q82

Please describe here in your own words any changes to your/your household's circumstances in the next three months that might make it easier or more difficult to continue owning your dog. **[free text]**
