# Supplementary material for: Lasting lockdown love? Problem behaviour and pandemic and non-pandemic related risk factors influencing the owner-dog relationship in a UK cohort of dogs reaching early adulthood
Source: PLoS One. 2025 Feb 12;20(2):e0316166. doi: 10.1371/journal.pone.0316166 (PMC11819559; doi:10.1371/journal.pone.0316166)
Supplement: S2 Appendix — (PDF) [file pone.0316166.s002.pdf]

S2 Appendix: Risk factor category frequency distributions and univariable associations with Monash Dog Owner Relationship Scale subscales "Perceived Emotional Closeness" and "Perceived Costs"

Table 1: Respondent demographics and univariable linear association with Perceived Emotional Closeness (PEC – Closeness) and Perceived Costs (PC – Costs) subscales of the Monash Dog Owner Relationship Scale reported by owners of dogs aged 21m, bought as puppies in the UK in July to December 2020.

Variable	Category	Number (%) n = 794	PEC^ β	PEC 95% CI Lower	Upper	PEC p-value*	PC[s] β	PEC 95% CI Lower	Upper	PC p-value*
**Respondent age (years)[1]**						**<0.001**				**0.110**
	18 – 24	35 (4.41)	+0.09	-2.29	+2.46		-2.39	-4.55	-0.22	
constant	25 – 34	153 (19.27)	*42.08*	*41.06*	*43.11*		*17.90*	*16.97*	*18.84*	
	35 – 44	155 (19.52)	-3.55	-4.99	-2.10		-0.84	-2.16	+0.47	
	45 – 54	216 (27.20)	-2.07	-3.41	-0.73		-1.52	-2.74	-0.30	
	55 – 64	144 (18.14)	-3.62	-5.09	-2.15		-1.22	-2.56	+0.12	
	65 – 74	79 (9.95)	-3.07	-4.83	-1.32		-1.79	-3.39	-0.19	
	75 +	11 (1.39)	-1.45	-5.41	+2.51		+0.10	-3.51	+3.71	
	No answer	1 (0.13)	+3.92	-8.80	+16.63		-8.90	+16.97	+18.83	
**Respondent gender[1]**						**0.007**				0.431
constant	Woman	719 (90.55)					*16.76*	*16.32*	*17.19*	
	Man	75 (9.45)	-2.15	-3.72	-0.59		+0.56	-0.84	+1.97	
	Other	0 (0.00)								
	Prefer not to say	0 (0.00)								
**UK Region[1]**						0.200				**0.004**
constant	East Midlands	48 (6.05)	*40.02*	*38.16*	*41.88*		*16.79*	*15.14*	*18.45*	
	East of England	106 (13.35)	-0.62	-2.87	+1.62		+0.72	-1.28	+2.71	
	London	88 (11.08)	-1.66	-3.97	+0.66		+1.02	-1.04	+3.07	
	North-East	31 (3.90)	+2.04	-0.93	+5.01		-2.02	-4.66	+0.63	
	North-West	63 (7.93)	+0.82	-1.65	+3.29		-2.33	-4.53	-0.13	
	Northern Ireland	4 (0.50)	+2.23	-4.48	+8.94		+4.21	-1.76	+10.18	
	Scotland	61 (7.68)	+0.13	-2.36	+2.61		-0.19	-2.40	+2.03	
	South-East	122 (15.37)	-0.93	-3.13	+1.27		+1.18	-0.76	+3.11	
	South-West	100 (12.59)	-0.32	-2.58	+1.94		-0.62	-2.64	+1.39	
	Wales	39 (4.91)	+1.11	-1.67	+3.89		-1.13	-3.60	+1.35	
	West Midlands	51 (6.42)	+0.84	-1.75	+3.43		+0.09	-2.22	+2.40	
	Yorkshire & the Humber	66 (8.31)	+0.40	-2.04	+2.85		-0.28	-2.45	+1.90	
	No answer	15 (1.89)	-1.15	-4.97	+2.66		+1.88	-1.52	+5.27	
**Employed in animal care sector[1]**						**0.079**				**0.028**
constant	No	737 (92.82)	*39.74*	*39.27*	*40.22*		*16.94*	*16.51*	*17.36*	
	Yes	57 (7.18)	+1.59	-0.18	+3.37		-1.78	-3.37	-0.18	
**Do not live with another adult[2]**						**0.006**				0.536
constant	No	686 (86.40)	*39.60*	*39.11*	*40.09*		*16.86*	*16.42*	*17.30*	
	Yes	108 (13.60)	+1.87	+0.54	+3.20		-0.38	-1.58	+0.82	
**Children in the home[1]**						**<0.001**				**0.051**
constant	No	552 (69.52)	*40.55*	*40.00*	*41.09*		*16.54*	*16.05*	*17.03*	
	Yes	242 (30.48)	-2.26	-3.24	-1.28		+0.89	0.00	+1.78	

[1] Data from Pandemic Puppies 2020 survey.

[2] Data from survey when same dogs reached 21 months old.

^ Possible scores for PEC scale: 10 -50; higher score correlates to increased perceived emotional closeness.

[s] Possible scores for PC scale: 9 -45; higher score correlates to increased perceived costs.

* p-value for F statistic, p<0.2 in bold.

Table 2: Respondent household characteristics and univariable linear association with Perceived Emotional Closeness (PEC – Closeness) and Perceived Costs (PC – Costs) subscales of the Monash Dog Owner Relationship Scale reported by owners of dogs aged 21m, bought as puppies in the UK in 2020.

Variable	Category	Number (%) n = 794	PEC[^] β	PEC 95% CI Lower	PEC 95% CI Upper	PEC p-value[*]	PC[s] β	PEC 95% CI Lower	PEC 95% CI Upper	PC p-value[*]
**Number of other dogs in home[1]**						**0.057**				**<0.001**
constant	0	571 (71.91)	*39.44*	*38.91*	*39.98*		*17.62*	*17.14*	*18.09*	
	1	150 (18.89)	+1.20	+0.02	+2.38		-2.81	-3.85	-1.77	
	2	41 (5.16)	+2.43	+0.35	+4.52		-2.57	-4.40	-0.74	
	3 or more	22 (2.77)	+1.69	-1.11	+4.49		-4.03	-6.49	-1.56	
	No answer	10 (1.26)	+0.96	-3.15	+5.06		-2.62	-6.23	+1.00	
**Dog in respondent's childhood home[1]**						**0.019**				**0.003**
constant	No	238 (29.97)	*39.02*	*38.19*	*39.86*		*17.78*	*17.03*	*18.53*	
	Yes	556 (70.03)	+1.19	+0.19	+2.19		-1.38	-2.28	-0.49	
**Previously owned or co-owned a dog as an adult[1]**						**0.071**				**<0.001**
constant	No	313 (39.42)	*39.30*	*38.57*	*40.03*		*18.41*	*17.77*	*19.05*	
	Yes	480 (60.45)	+0.94	0.00	+1.88		-2.64	-3.46	-1.81	
	No answer	1 (0.13)	-7.30	-20.22	+5.62		-5.41	-16.75	+5.94	
**Work location when dog 21 months old[2]**						**0.014**				**0.079**
constant	Home	188 (23.68)	*39.84*	*38.90*	*40.77*		*17.50*	*16.66*	*18.34*	
	Away from home	169 (21.28)	+1.48	+0.12	+2.85		-1.55	-2.77	-0.32	
	Home and away	248 (31.23)	-0.41	-1.65	+0.84		-0.29	-1.41	+0.82	
	Unemployed	27 (3.40)	+0.28	-2.37	+2.92		-0.87	-3.25	+1.51	
	Retired	162 (20.40)	-0.87	-2.24	+0.51		-1.19	-2.42	+0.05	
**Other adult works away from home[2]**						**<0.001**				0.259
constant	No	555 (69.90)	*39.34*	*38.80*	*39.89*		*16.96*	*16.47*	*17.46*	
	Yes	239 (30.10)	+1.71	+0.72	+2.71		-0.52	-4.41	+0.38	
**Covid-19 affected finances[2]**						**<0.001**				0.387
constant	Same	515 (64.86)	*39.00*	*38.44*	*39.56*		*16.63*	*16.12*	*17.14*	
	Worse	133 (16.75)	+2.25	+1.01	+3.49		+0.80	-0.34	+1.92	
	Better	100 (12.59)	+2.94	+1.54	+4.33		-0.05	-1.32	+1.22	
	Rather not say	23 (2.90)	+1.09	-1.63	+3.80		-0.15	-2.62	+2.32	
	Not sure	18 (2.27)	+2.61	-0.11	+5.32		+1.93	-0.54	+4.41	
	No answer	5 (0.63)								
**Anticipated changes to household circumstances in next 3 months[2]**						0.346				**<0.001**
constant	No	692 (87.15)	*39.71*	*39.22*	*40.20*		*16.48*	*16.04*	*16.91*	
	Yes, easier to own dog	43 (5.42)	+1.78	-0.25	+3.81		+1.96	+0.16	+3.76	
	Yes, harder to own dog	52 (6.55)	+0.65	-1.21	+2.51		+3.66	+2.01	+5.30	
	No answer	7 (0.88)	+0.57	-4.34	+5.48		-1.76	-6.11	+2.58	

[1] Data from Pandemic Puppies 2020 survey.

[2] Data from survey when same dogs reached 21 months old.

^ Possible scores for PEC scale: 10 -50; higher score correlates to increased perceived emotional closeness.

[s] Possible scores for PC scale: 9 -45; higher score correlates to increased perceived costs.

* p-value for F statistic, p<0.2 in bold.

Table 3: Puppy characteristics and univariable linear association with Perceived Emotional Closeness (PEC – Closeness) and Perceived Costs (PC – Costs) subscales of the Monash Dog Owner Relationship Scale reported by owners of dogs aged 21m, bought as puppies in the UK in July to December 2020.

Variable	Category	Number (%) n = 794	PEC^ β	PEC 95% CI Lower	Upper	PEC p-value*	PCˢ β	PEC 95% CI Lower	Upper	PC p-value*
**Typical adult bodyweight (kg)** [1]						**0.001**				0.532
constant	<10	148 (18.64)	*41.33*	*40.28*	*42.38*		*16.19*	*15.24*	*17.14*	
	10 – <20	309 (38.92)	-1.71	-2.99	-0.43		+0.95	-0.21	+2.11	
	20 – <30	180 (22.67)	-2.94	-4.36	-1.52		+0.74	-0.54	+2.03	
	30 – <40	114 (14.36)	-0.67	-2.27	+0.92		+0.08	-1.36	-1.53	
	40 and over	14 (1.76)	+0.81	-2.77	+4.39		+1.24	-2.00	+4.48	
	Breed type of varied size	29 (3.65)	-1.64	-4.24	+0.96		+1.26	-1.10	+3.62	
**Kennel Club Breed Group** [1]						0.383				**0.080**
constant	Not KC recognised	250 (31.49)	*39.55*	*38.73*	*40.37*		*17.50*	*16.77*	*18.23*	
	Gundog	242 (30.48)	-0.26	-1.42	+0.91		-1.02	-2.06	+0.02	
	Hound	73 (9.19)	+0.27	-1.45	+1.99		+0.08	-1.46	+1.62	
	Pastoral	58 (7.30)	+1.21	-0.67	+3.09		-0.05	-1.73	+1.64	
	Terrier	69 (8.69)	+1.42	-0.33	+3.18		-2.28	-3.85	-0.71	
	Toy	19 (2.39)	+1.93	-1.15	+5.00		-1.18	-3.93	+1.57	
	Utility	53 (6.68)	+0.96	-0.99	+2.92		-1.51	-3.26	+0.23	
	Working	30 (3.78)	+1.05	-1.44	+3.55		-1.40	-3.63	+0.84	
**Breed designation** [1]						0.357				**0.042**
	Crossbred	32 (4.03)	+1.04	-1.31	+3.39		-0.32	-2.43	+1.78	
constant	Purebred	551 (69.40)	*39.96*	*39.41*	*40.51*		*16.51*	*16.02*	*17.0*	
	Designer Crossbred	211 (26.57)	-0.55	-1.59	+0.50		+1.17	+0.23	+2.10	
**Pug, French bulldog, or English bulldog** [1]						**0.176**				0.653
constant	No	781 (98.36)	*39.82*	*39.35*	*40.28*		*16.82*	*16.41*	*17.24*	
	Yes	13 (1.64)	+2.49	-1.12	+6.10		-0.74	-3.99	+2.50	
**Sex** [1]						0.649				**0.179**
constant	Male	420 (52.90)	*40.03*	*39.40*	*40.66*		*17.17*	*16.60*	*17.73*	
	Female	373 (46.98)	-0.37	-1.29	+0.55		-0.75	-1.58	+0.07	
	No answer	1 (0.13)	+2.97	-9.98	+15.92		-3.17	-14.76	+8.43	
**Neutered prior to 21m survey** [2]						**0.012**				**0.010**
constant	No	326 (41.06)	*40.56*	*39.85*	*41.27*		*16.16*	*15.52*	*16.80*	
	Yes	468 (58.94)	-1.20	-2.13	-0.27		1.10	+0.27	+1.93	
**Birth month (year 2020)** [1]						**0.157**				0.200
constant	May	88 (11.08)	*40.67*	*39.30*	*42.05*		*16.38*	*15.14*	*17.61*	
	June	109 (13.73)	-0.10	-1.95	+1.75		-0.18	-1.84	+1.48	
	July	139 (17.51)	-0.17	-1.92	+1.59		-0.02	-1.60	+1.55	
	August	161 (20.28)	-1.11	-2.82	+0.60		+0.56	-0.98	+2.09	
	September	178 (22.42)	-1.43	-3.12	+0.25		+0.77	-0.74	+2.27	
	October	113 (14.23)	-1.71	-3.54	+0.13		+1.42	-0.22	+3.07	
	November	6 (0.76)	+2.33	-3.11	+7.77		-3.21	-8.09	+1.68	

[1] Data from Pandemic Puppies 2020 survey.

[2] Data from survey when same dogs reached 21 months old.

^ Possible scores for PEC scale: 10 -50; higher score correlates to increased perceived emotional closeness.

ˢ Possible scores for PC scale: 9 -45; higher score correlates to increased perceived costs.

* p-value for F statistic, p<0.2 in bold.

Table 4: Puppy acquisition motivation and preparation univariable linear association with Perceived Emotional Closeness (PEC – Closeness) and Perceived Costs (PC – Costs) subscales of the Monash Dog Owner Relationship Scale reported by owners of dogs aged 21m, bought as puppies in the UK in July to December 2020.

Variable	Category	Number (%) n = 794	PEC^ β	PEC 95% CI Lower	PEC 95% CI Upper	PEC p-value*	PC[s] β	PEC 95% CI Lower	PEC 95% CI Upper	PC p-value*
**Companionship for myself [1]**						**<0.001**				0.571
constant	No	264 (33.25)	*38.66*	*37.87*	*39.45*		*16.64*	*15.93*	*17.35*	
	Yes	530 (66.75)	+1.79	+0.82	+2.75		+0.25	-0.62	+1.13	
**Companionship for other people in household, not respondent [1]**						**<0.001**				**0.002**
constant	No	708 (89.17)	*40.28*	*39.80*	*40.75*		*16.58*	*16.14*	*17.01*	
	Yes	86 (10.83)	-3.87	-5.32	-2.42		-2.13	-3.45	-0.82	
**Working dog for specific role [1]**						**0.030**				**0.001**
constant	No	747 (94.08)	*39.73*	*39.26*	*40.20*		*16.98*	*16.55*	*17.40*	
	Yes	47 (5.92)	+2.14	+0.20	+4.08		-2.83	-4.56	-1.09	
**Covid-19 pandemic influenced decision to acquire[1]**						0.613				**<0.001**
constant	No	446 (56.17)	*40.00*	*39.39*	*40.61*		*15.60*	*15.07*	*16.13*	
	Yes	321 (40.43)	-0.26	-1.20	+0.69		+2.48	+1.66	+3.30	
	Not sure	26 (3.27)	-1.19	-3.75	+1.38		+5.95	+3.73	+8.18	
	No answer	1 (0.13)								
**Prior research[1]**						0.327				**<0.001**
	No	15 (1.89)	-0.53	-3.92	+2.86		-1.43	-4.41	+1.55	
constant	Yes	462 (58.19)	*39.53*	*38.93*	*40.13*		*17.83*	*17.30*	*18.36*	
	No, already experienced	315 (39.67)	0.85	-0.10	1.79		-2.54	-3.37	-1.71	
	No answer	2 (0.25)	-1.03	-10.19	+8.13		+4.67	-3.37	+12.71	
**Decision to acquisition[1]**						**0.004**				0.411
	<1w	15 (1.89)	+2.72	-0.70	+6.12		-1.00	-4.06	+2.07	
	1w – 1m	59 (7.43)	+2.33	+0.51	+4.16		-1.17	-2.81	+0.48	
	1 – 6m	410 (51.64)	+0.86	-0.11	+1.82		-0.27	-1.15	+0.60	
constant	>6m	309 (38.92)	*39.21*	*38.48*	*39.94*		*17.06*	*16.41*	*17.72*	
	No answer	1 (0.13)	-16.21	-29.06	-3.36		-8.06	-19.68	+3.55	

[1] Data from Pandemic Puppies 2020 survey.

[2] Data from survey when same dogs reached 21 months old.

^ Possible scores for PEC scale: 10 -50; higher score correlates to increased perceived emotional closeness.

[s] Possible scores for PC scale: 9 -45; higher score correlates to increased perceived costs.

* p-value for F statistic, p<0.2 in bold.

Table 4: Puppy acquisition practices univariable linear association with Perceived Emotional Closeness (PEC – Closeness) and Perceived Costs (PC – Costs) subscales of the Monash Dog Owner Relationship Scale reported by owners of dogs aged 21m, bought as puppies in the UK in July to December 2020.

Variable	Category	Number (%) n = 794	PEC^ β	PEC 95% CI Lower	PEC 95% CI Upper	PEC p-value*	PC[s] β	PEC 95% CI Lower	PEC 95% CI Upper	PC p-value*
**Acquired first choice breed[1]**						0.481				**0.105**
	No	68 (8.56)	-0.86	-2.50	+0.79		+1.74	+0.26	+3.21	
constant	Yes	670 (84.38)	*39.96*	*39.46*	*40.46*		*16.68*	*16.23*	*17.13*	
	No answer	1 (0.13)	+7.04	-5.90	+19.98		-4.68	-16.23	+17.12	
**Price category (£)[1]**						**0.193**				**0.033**
	<500	10 (1.26)	+5.18	+0.93	+9.44		-2.79	-6.60	+1.01	
constant	500 – 999	111 (13.98)	*40.22*	*38.99*	*41.44*		*15.79*	*14.70*	*16.89*	
	1000 – 1499	165 (20.78)	-0.50	-2.08	+1.09		0.35	-1.06	+1.77	
	1500 – 1999	174 (21.91)	-0.31	-1.88	+1.25		+1.56	+0.16	+2.96	
	2000 – 2999	287 (36.15)	-0.57	-2.01	+0.87		+1.56	+0.27	+2.85	
	Prefer not to say	43 (5.42)	-1.03	-3.35	+1.29		+1.09	-0.98	+3.16	
	Can't remember	1 (0.13)	+1.78	-4.78	+8.35		+2.21	-3.67	+8.08	
	No answer	3 (0.38)								
**Collected inside breeder's home[1]**						0.211				**0.108**
constant	No	292 (36.78)	*39.47*	*38.72*	*40.23*		*17.25*	*16.57*	*17.92*	
	Yes	502 (63.22)	+0.61	-0.34	+1.56		-0.70	-1.55	+0.15	
**Puppy seen with mother at collection[1]**						**0.050**				**0.001**
constant	No	166 (20.91)	*39.24*	*38.24*	*40.24*		*18.33*	*17.43*	*19.22*	
	Yes	626 (78.84)	+0.81	-0.32	+1.93		-1.91	-2.92	-0.91	
	No answer	2 (0.25)	-8.74	-17.91	+0.43		-3.33	-11.51	+4.86	
**Microchip provided by breeder[1]**						0.887				0.798
	No	33 (4.16)	+0.26	-2.05	+2.56		+0.25	-1.82	+2.31	
constant	Yes	753 (94.84)	*39.84*	*39.36*	*40.31*		*16.81*	*16.39*	*17.23*	
	No answer	8 (1.01)	+1.04	-3.56	+5.64		-1.31	-5.44	+2.81	
**Passport provided by breeder[1]**						**0.173**				0.634
constant	No	464 (58.44)	*40.03*	*39.53*	*40.53*		*17.02*	*16.48*	*17.56*	
	Not applicable	214 (26.95)								
	Yes	44 (5.54)	-0.76	-2.77	1.25		+0.83	-0.97	+2.64	
	Not sure	28 (3.53)	-1.45	-3.05	0.15		+0.28	-1.16	+1.71	
	No answer	44 (5.54)								

[1] Data from Pandemic Puppies 2020 survey.

[2] Data from survey when same dogs reached 21 months old.

^ Possible scores for PEC scale: 10 -50; higher score correlates to increased perceived emotional closeness.

[s] Possible scores for PC scale: 9 -45; higher score correlates to increased perceived costs.

* p-value for F statistic, p<0.2 in bold.

Table 6: Dog management and health univariable linear association with Perceived Emotional Closeness (PEC – Closeness) and Perceived Costs (PC – Costs) subscales of the Monash Dog Owner Relationship Scale reported by owners of dogs aged 21m, bought as puppies in the UK in July to December 2020.

Variable	Category	Number (%) n = 794	PEC^ β	PEC 95% CI Lower	Upper	PEC p-value*	PC[s] β	PEC 95% CI Lower	Upper	PC p-value*
**Primary carer of dog in 2020[1]**						**0.083**				0.721
	No	2 (0.25)	+0.85	-8.28	+9.99		-0.83	-9.06	+7.39	
constant	Yes, sole	454 (57.18)	*40.15*	*39.54*	*40.75*		*16.83*	*16.29*	*17.38*	
	Yes, shared within household	323 (40.68)	-0.52	-1.46	+0.42		-0.14	-0.98	+0.71	
	Yes, shared outside household	15 (1.89)	-4.21	-7.60	-0.83		+1.63	-1.41	+4.68	
**Change in who involved in dog's care from 2020 to 21 months old[2]**						0.897				**<0.001**
constant	No	206 (25.94)	*39.75*	*38.85*	*40.65*		*18.15*	*17.35*	*18.95*	
	Yes	583 (73.43)	+0.14	-0.91	+1.19		-1.82	-2.76	-0.89	
	No answer	5 (0.63)	+1.25	-4.60	+7.11		-0.55	-5.76	+4.66	
**Puppy left alone >4h in 2020[1]**						0.291				0.444
constant	No	782 (98.49)	*39.82*	*39.35*	*40.28*		*16.78*	*16.37*	*17.19*	
	Yes	11 (1.39)	+2.28	-1.65	+6.20		+2.22	-1.30	+5.74	
	No answer	1 (0.13)	+7.18	-5.74	+20.11		-1.78	-13.37	+17.19	
**Dog left alone >4h at 21 months old[2]**						**0.001**				0.532
constant	No	690 (86.90)	*39.56*	*39.07*	*40.05*		*16.86*	*16.42*	*17.30*	
	Yes	104 (13.10)	+2.25	+0.89	+3.60		-0.39	-1.61	+0.83	
**Take dog if go out to work at 21 months old[2]**						**0.016**				**0.015**
constant	Don't go out to work	348 (43.83)	*38.97*	*38.29*	*39.66*		*16.94*	*16.33*	*17.55*	
	Someone else does	6 (0.76)								
	No	359 (45.21)	+1.48	+0.52	+2.45		+0.21	-0.65	+1.08	
	Always	13 (1.64)	+2.10	-1.53	+5.73		-1.79	-5.04	+1.47	
	At least half of workdays	22 (2.77)	+2.21	-0.62	+5.03		-3.40	-5.93	-0.86	
	Less than half of workdays	42 (5.29)	+1.79	-0.31	+3.89		-2.11	-3.99	-0.23	
	No answer	4 (0.50)	+4.27	-2.19	+10.74		+1.31	-4.49	+7.11	
**Puppy classes at <16wo[1]**						0.464				0.216
constant	No	455 (57.30)	*40.00*	*39.40*	*40.61*		*16.58*	*16.04*	*17.13*	
	Yes	339 (42.70)	-0.35	-1.27	+0.58		+0.52	-0.31	+1.36	
**Adult training classes to 21mo[2]**						0.483				0.715
constant	No	431 (54.28)	*39.66*	*39.03*	*40.28*		*16.65*	*16.09*	*17.21*	
	In person	332 (41.81)	+0.52	-0.43	+1.46		+0.33	-0.51	+1.18	
	Online	31 (3.90)	-0.43	-2.83	+1.97		+0.44	-1.71	+2.60	
**Training method[2]**						**0.015**				**0.005**
constant	Rewards-only	139 (17.51)	*39.50*	*38.41*	*40.59*		*15.96*	*14.99*	*16.94*	
	Aversive only	0 (0.00)								
	Rewards & 1 aversive	162 (20.40)	+1.61	+0.13	+3.10		+0.19	-1.14	+1.52	
	Rewards & aversive	470 (59.19)	+0.16	-1.08	+1.41		+1.43	+0.32	+2.54	
	No answer	23 (2.90)	-2.28	-5.17	+0.62		-1.44	-4.04	+1.15	
**Dog has ongoing health problem[2]**						0.460				**0.131**
constant	No	728 (91.69)	*39.89*	*39.41*	*40.37*		*16.69*	*16.26*	*17.12*	
	Yes	65 (8.19)	-0.31	-1.98	+1.37		+1.46	-0.04	+2.96	
	No answer	1 (0.13)	-7.89	-20.83	+5.05		-3.69	-15.28	+7.89	

[1] Data from Pandemic Puppies 2020 survey.
[2] Data from survey when same dogs reached 21 months old.
^ Possible scores for PEC scale: 10 -50; higher score correlates to increased perceived emotional closeness.
[s] Possible scores for PC scale: 9 -45; higher score correlates to increased perceived costs.
* p-value for F statistic, p<0.2 in bold.

Table 7: Dog problematic behaviour univariable linear association with Perceived Emotional Closeness (PEC – Closeness) and Perceived Costs (PC – Costs) subscales of the Monash Dog Owner Relationship Scale reported by owners of dogs aged 21m, bought as puppies in the UK in July to December 2020.

Variable	Category	Number (%) n = 794	PEC[^] β	PEC 95% CI Lower	PEC 95% CI Upper	PEC p-value[*]	PC[s] β	PEC 95% CI Lower	PEC 95% CI Upper	PC p-value[*]
**Control problem:** Number of different problem behaviours reported in 3 months prior to survey[2]						**0.024**				**<0.001**
constant	0	145 (18.26)	*41.49*	*40.34*	*42.64*		*15.02*	*14.01*	*16.04*	
	1	244 (30.73)	-1.99	-3.41	-0.58		+1.40	+0.15	+2.65	
	2	274 (34.51)	-1.60	-2.99	-0.21		+1.83	+0.61	+3.06	
	3	131 (16.50)	-2.31	-3.92	-0.70		+4.15	+2.73	+5.57	
	No answer	20 (2.52)	-3.39	-6.49	-0.29		+1.48	-1.26	+4.22	
**Attention seeking:** Number of different problem behaviours reported in 3 months prior to survey[2]						0.303				**<0.001**
constant	0	174 (21.91)	*39.79*	*38.81*	*40.77*		*14.64*	*13.78*	*15.50*	
	1	247 (31.11)	-0.30	-1.57	+0.98		+2.24	+1.12	+3.36	
	2	204 (25.69)	+0.30	-1.03	+1.64		+3.23	+2.06	+4.41	
	3	92 (11.59)	+0.97	-0.70	+2.63		+2.89	+1.43	+4.36	
	4	26 (3.27)	+1.21	-1.31	+3.72		+4.23	+2.02	+6.45	
	5	5 (0.63)								
	No answer	46 (5.79)	-1.42	-3.57	+0.72		+2.45	+0.57	+4.33	
**Aggression:** Number of different problem behaviours reported in 3 months prior to survey[2]						**0.121**				**<0.001**
constant	0	596 (75.06)	*39.99*	*39.46*	*40.52*		*16.47*	*16.00*	*16.94*	
	1	138 (17.38)	-0.48	-1.69	+0.74		+0.41	-0.66	+1.49	
	2	37 (4.66)	+0.98	-1.20	+3.17		+2.29	+0.35	+4.22	
	3	10 (1.26)	-3.30	-7.60	+0.62		+7.84	+4.64	+11.04	
	4	3 (0.38)								
	No answer	10 (1.26)	-3.49	-7.60	+0.62		+2.43	-1.21	+6.07	
**Fear/avoidance:** Number of different problem behaviours reported in 3 months prior to survey[2]						**0.174**				**<0.001**
constant	0	455 (57.30)	*39.51*	*38.90*	*40.11*		*16.29*	*15.75*	*16.83*	
	1	198 (24.94)	+0.95	-0.15	+2.05		+0.34	-0.64	+1.32	
	2	93 (11.71)	+0.92	-0.55	+2.39		+1.84	+0.53	+3.14	
	3	28 (3.53)	+1.02	-1.27	+3.31		+3.92	+1.88	+5.95	
	4	6 (0.76)								
	No answer	14 (1.76)	-2.37	-5.87	+1.13		+2.85	-0.26	+5.96	
**Reaction to familiar people:** Number of different problem behaviours reported in 3 months prior to survey[2]						0.909				**<0.001**
constant	0	746 (93.95)	*39.87*	*39.40*	*40.35*		*16.56*	*16.15*	*16.98*	
	1	33 (4.16)	-0.35	-3.99	+4.69		+4.80	+2.92	+6.67	
	2	6 (0.76)								
	No answer	9 (1.13)	+0.35	-3.99	+1.69		+0.88	-2.95	4.71	
**Reaction to other dogs:** Number of different problem behaviours reported in 3 months prior to survey[2]						**0.199**				**0.002**
constant	0	615 (77.46)	*39.75*	*39.23*	*40.27*		*16.39*	*15.92*	*16.85*	
	1	112 (14.11)	+0.71	-0.62	+2.03		+1.58	+0.39	+2.76	
	2	58 (7.30)	+0.70	-1.07	+2.47		+2.27	+0.69	+3.85	
	No answer	9 (1.13)	-3.75	-8.08	+0.59		+2.83	-1.03	+6.70	
**Abnormal repetitive behaviours:** Number of different behaviours reported in 3 months prior to survey[2]						0.834				**<0.001**
constant	0	498 (62.72)	*39.70*	*39.12*	*40.28*		*16.28*	*15.76*	*16.79*	
	1	198 (24.94)	+0.46	-0.62	+1.55		+0.89	-0.07	+1.86	
	2	72 (9.07)	+0.46	-1.18	+2.09		+2.87	+1.42	+4.32	
	3	17 (2.14)	0.07	-2.53	+2.68		+1.41	-0.90	+3.73	
	4	6 (0.76)								
	5	3 (0.38)								
**Separation related behaviours[2]**						0.469				**<0.001**
constant	No	496 (62.47)	*39.85*	*39.27*	*40.43*		*15.95*	*15.44*	*16.46*	
	Yes	222 (27.96)	-0.25	-1.30	+0.80		+2.84	+1.92	+3.75	
	No answer	76 (9.57)	+0.82	-0.77	+2.41		+0.67	-0.72	+2.06	

[1] Data from Pandemic Puppies 2020 survey.
[2] Data from survey when same dogs reached 21 months old.
[^] Possible scores for PEC scale: 10 -50; higher score correlates to increased perceived emotional closeness.
[s] Possible scores for PC scale: 9 -45; higher score correlates to increased perceived costs.
* p-value for F statistic, p<0.2 in bold.
