# Supplementary material for: Lasting lockdown love? Problem behaviour and pandemic and non-pandemic related risk factors influencing the owner-dog relationship in a UK cohort of dogs reaching early adulthood
Source: PLoS One. 2025 Feb 12;20(2):e0316166. doi: 10.1371/journal.pone.0316166 (PMC11819559; doi:10.1371/journal.pone.0316166)
Supplement: S3 Appendix — (PDF) [file pone.0316166.s003.pdf]

S3 Appendix: Monash Dog Owner Relationship Scale item frequency and subscale score distributions

Table 1: Frequency of responses to Monash Dog Owner Relationship Scale items reported by owners of 21-month-old dogs acquired as puppies in July to December 2020 in the UK (n=794).

Subscale	Item	Frequency (%) of responses				
		1	2	3	4	5
**Dog-Owner Interaction**						
	How often do you play games with your dog?[a]	1 (0.13)	1 (0.13)	6 (0.76)	81 (10.2)	705 (88.79)
	How often do you take your dog to visit people?[a]	36 (4.53)	153 (19.27)	220 (27.71)	301 (37.91)	84 (10.58)
	How often do you give your dog food treats?[a]	3 (0.38)	6 (0.76)	6 (0.76)	74 (9.32)	705 (88.79)
	How often do you kiss your dog?[a]	77 (9.70)	7 (0.88)	15 (1.89)	52 (6.55)	643 (80.98)
	How often do you take your dog in the car?[a]	10 (1.26)	52 (6.55)	164 (20.65)	355 (44.71)	213 (26.83)
	How often do you hug your dog?[a]	15 (1.89)	3 (0.38)	7 (0.88)	27 (3.40)	742 (93.45)
	How often do you buy your dog presents?[a]	16 (2.02)	245 (30.86)	307 (38.66)	147 (18.51)	79 (9.95)
	How often do you have your dog with you while relaxing, i.e., watching TV?[a]	3 (0.38)	0 (0.00)	1 (0.13)	8 (1.01)	782 (98.49)
	How often do you groom your dog?[a]	25 (3.15)	133 (16.75)	211 (26.57)	271 (34.13)	154 (19.4)
**Perceived Emotional Closeness (PEC)**						
	My dog helps me get through tough times.[lk]	4 (0.40)	7 (0.88)	84 (10.58)	251 (31.61)	448 (56.42)
	My dog is there whenever I need to be comforted.[lk]	4 (0.50)	11 (1.39)	75 (9.45)	242 (30.48)	462 (58.19)
	I would like to have my dog near me all the time.[lk]	7 (0.88)	82 (10.33)	221 (27.83)	249 (31.36)	235 (29.60)
	My dog provides me with constant companionship.[lk]	3 (0.38)	11 (1.39)	58 (7.3)	317 (39.92)	405 (51.01)
	If everyone else left me my dog would still be there for me.[lk]	2 (0.25)	6 (0.76)	87 (10.96)	247 (31.11)	452 (56.93)
	My dog gives me a reason to get up in the morning.[lk]	12 (1.51)	60 (7.56)	222 (27.96)	227 (28.59)	273 (34.38)
	I wish my dog and I never had to be apart.[lk]	17 (2.14)	185 (23.30)	261 (32.87)	168 (21.16)	163 (20.53)
	My dog is constantly attentive to me.[lk]	6 (0.76)	120 (15.11)	200 (25.19)	287 (36.15)	181 (22.80)
	How often do you tell your dog things you don't tell anyone else? [secret][a]	372 (46.85)	9 (1.13)	45 (5.67)	160 (20.15)	208 (26.20)
	How traumatic do you think it will be for you when your dog dies?[t]	2 (0.25)	2 (0.25)	15 (1.89)	175 (22.04)	600 (75.57)
**Perceived Costs (PC)**						
	How often do you feel that looking after your dog is a chore?[a]	581 (73.17)	39 (4.91)	98 (12.34)	62 (7.81)	14 (1.76)

	It is annoying that I sometimes have to change my plans because of my dog.[lk]	180 (22.67)	284 (35.77)	187 (23.55)	130 (16.37)	13 (1.64)
	It bothers me that my dog stop me doing things I enjoyed doing before I owned it.[lk]	285 (35.89)	330 (41.56)	117 (14.74)	52 (6.55)	10 (1.26)
	There are major aspects of owing a dog I don't like.[lk]	382 (48.11)	295 (37.15)	70 (8.82)	40 (5.04)	7 (0.88)
	How often does your dog stop you doing things you want to?[a]	360 (45.34)	134 (16.88)	184 (23.17)	92 (11.59)	24 (3.02)
	My dog make too much mess.[lk]	388 (48.87)	291 (36.65)	89 (11.21)	22 (2.77)	4 (0.50)
	My dog costs too much money.[lk]	340 (42.82)	300 (37.78)	130 (16.37)	20 (2.52)	4 (0.50)
	How hard is it to look after your dog?[h]	145 (18.26)	306 (38.54)	284 (35.77)	53 (6.68)	6 (0.76)
	How often do you feel that having a dog is more trouble than it is worth?[a]	714 (89.92)	25 (3.15)	34 (4.28)	15 (1.89)	6 (0.76)

[a] responses are integers 1-5 on an adjectival scale from never=1 to frequent=5

[lk] responses are integers 1-5 on a Likert scale strongly disagree =1 to strongly agree = 5
[t] responses are integers 1-5 on a Likert-like scale very untraumatic =1 to very traumatic = 5
[h] responses are integers 1-5 on a Likert-like scale very easy =1 to very hard = 5

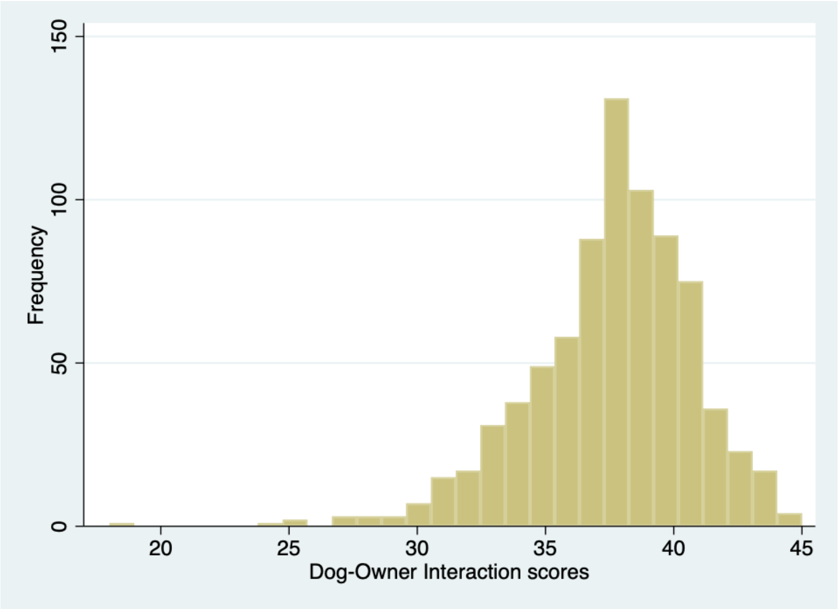

Figure 1: Frequency of Dog-Owner Interaction scores reported by owners of 21-month-old dogs acquired as puppies in July to December 2020 in the UK (n=794).

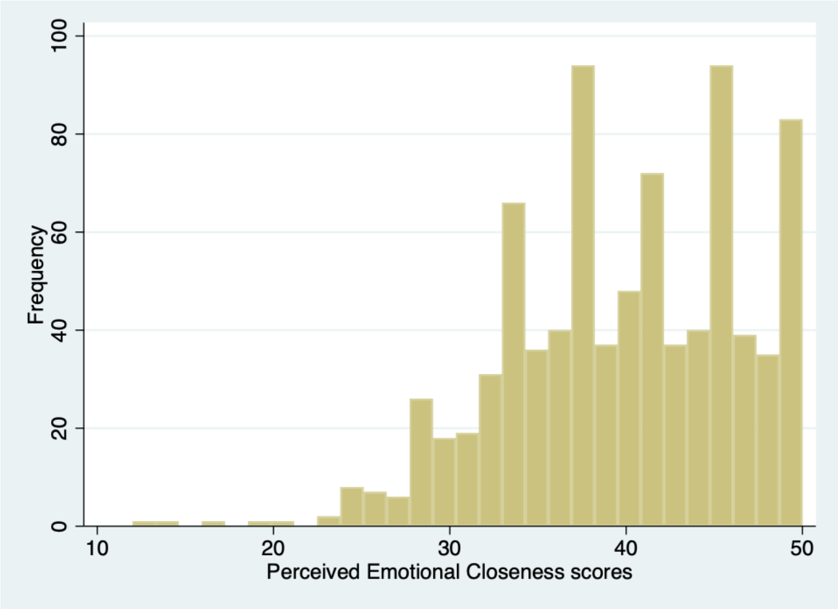

Figure 2: Frequency of Perceived Emotional Closeness scores reported by owners of 21-month-old dogs acquired as puppies in July to December 2020 in the UK (n=794).

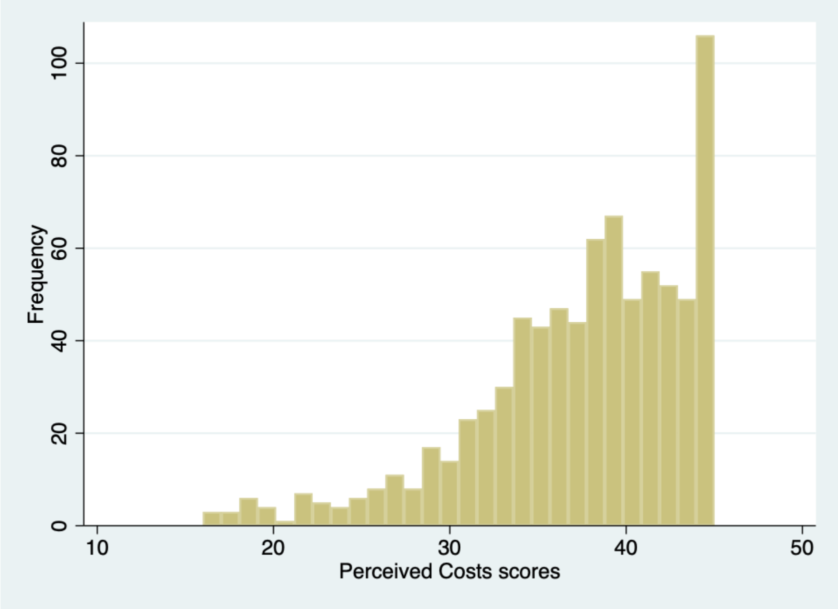

Figure 3: Frequency of Perceived Costs scores reported by owners of 21-month-old dogs acquired as puppies in July to December 2020 in the UK (n=794).

Appendix 4: Full mixed linear regression models for Closeness and Costs, with effect sizes and regression diagnostics

Table 1: Mixed linear regression model for Perceived Emotional Closeness (PEC – Closeness) between owners and their 21-month old dogs acquired as puppies in July to December 2020 in the UK, measured by the PEC subscale of the Monash Dog-Owner Relationship Scale (n=794).

			95% Confidence Interval			Effect size		
		Multi-				Cohen's	95% CI	
**Variable**	**Category *(n)***	**variable β**	**Lower**	**Upper**	**p-value***	**d_s#**	**Lower**	**Upper**
	Constant:	***39.14***	***37.10***	***41.17***	***<0.001***			
**Respondent Age (years)**[1]								
	18 – 24 *(n=35)*	+0.75	-1.44	+2.94	0.503			
	25 – 34 *(n=153)*	base						
	**35 – 44** *(n=155)*	**-2.02**	**-3.46**	**-0.60**	**0.005**	**-0.31**	**-0.54**	**-0.09**
	45 – 54 *(n=216)*	-0.64	-2.00	+0.71	0.353			
	**55 – 64** *(n=144)*	**-2.65**	**-4.22**	**-1.08**	**0.001**	**-0.41**	**-0.64**	**-0.18**
	65 – 74 *(n=79)*	-2.03	-4.28	+0.22	0.077			
	75 + *(n=11)*	-0.12	-4.06	3.82	0.952			
	No response *(n=1)*	3.82	-7.62	15.26	0.513			
**Respondent gender**[1]								
	Woman *(n=719)*	base						
	**Man** *(n=75)*	**-1.61**	**-3.02**	**-0.20**	**0.026**	**-0.24**	**-0.48**	**-0.01**
**Dog's typical adult bodyweight by breed (kg)** [1]								
	<10 *(n=148)*	base						
	**10 – <20** *(n=309)*	**-2.00**	**-3.15**	**-0.84**	**0.001**	**-0.31**	**-0.50**	**-0.11**
	**20 – <30** *(n=180)*	**-3.35**	**-4.67**	**-2.02**	**<0.001**	**-0.53**	**-0.75**	**-0.30**
	30 – <40 *(n=114)*	-1.09	-2.58	+0.40	0.152			
	40 and over *(n=14)*	-0.39	-3.64	+2.85	0.812			
	Breed type of varied size *(n=29)*	-2.10	-4.46	+0.26	0.081			
**Respondent lives with another adult person**[2]								
	Yes *(n=686)*	base						
	**No** *(n=108)*	**+1.46**	**+0.10**	**+2.81**	**0.035**	**+0.22**	**+0.02**	**+0.43**
**Children in the home**[1]								
	No *(n= 552)*	base						
	**Yes** *(n=242)*	**-1.88**	**-2.95**	**-0.81**	**0.001**	**-0.29**	**-0.44**	**-0.14**
**Previously owned or co-owned a dog as an adult**[1]								
	No *(n=313)*	base						
	**Yes** *(n=480)*	**+1.47**	**+0.53**	**+2.41**	**0.002**	**+0.26**	**+0.12**	**+0.41**
	Not answered *(n=1)*	-4.48	-15.91	+6.94	0.442			
**Work location when dog 21 months old**[2]								
	Work from home *(n=188)*	base						
	Away from home *(n=169)*	+0.11	-1.34	+1.58	0.874			
	**Home and away** *(n=248)*	**-1.48**	**-2.81**	**-0.16**	**0.028**	**-0.23**	**-0.43**	**-0.02**
	Unemployed *(n=27)*	-0.46	-2.99	+2.07	0.721			
	Retired *(n=162)*	-0.57	-2.30	+1.15	0.514			
**Other adult works away from home**[2]								
	No, or no other adult *(n=555)*	base						
	**Yes** *(n=239)*	**+1.26**	**+0.31**	**+2.21**	**0.009**	**+0.19**	**+0.04**	**+0.34**
**Owner takes dog with them if go out to work (dog 21 months old)**[2]								
	Don't go out or other adult takes dog *(n=354)*	base						
	Always *(n=13)*	+2.79	-0.74	+6.32	0.122			
	At least half of workdays *(n=22)*	+2.30	-0.39	+4.99	0.093			
	Less than half of workdays *(n=42)*	+1.17	-0.93	+3.27	0.275			
	**Never** *(n=359)*	**+1.43**	**+0.17**	**2.69**	**0.026**	**+0.22**	**+0.07**	**+0.37**
	Not answered *(n=4)*	+5.01	-0.80	10.82	0.091			
**Covid-19 affected finances**[2]								
	No *(n=515)*	base						
	**Better** *(n=133)*	**+1.86**	**+0.71**	**+3.00**	**0.001**	**+0.28**	**+0.09**	**+0.48**
	**Worse *(n=100)***	**+2.34**	**+1.03**	**+3.65**	**<0.001**	**+0.36**	**+0.14**	**+0.57**

Rather not say *(n=23)*	**+1.02**	-1.47	+3.51	0.421			
Not sure *(n=18)*	+2.07	-0.72	+4.86	0.146			
No answer *(n=5)*	+4.30	-0.90	+9.49	0.105			
**Companionship for myself** [1]							
No *(n=264)*	base						
**Yes** *(n=530)*	**+1.76**	**+0.86**	**+2.67**	**<0.001**	**+0.27**	**+0.12**	**+0.42**
**Working dog for specific role** [1]							
No *(n=747)*	base						
**Yes** *(n=47)*	**+2.12**	**+0.28**	**+3.95**	**0.024**	**+0.32**	**+0.03**	**+0.62**
**Decision to acquisition**[1]							
**Less than a week** *(n=15)*	**+4.06**	**+1.00**	**+7.13**	**0.009**	**+0.62**	**+0.10**	**+1.14**
**Week to month** *(n=59)*	**+3.14**	**+1.48**	**+4.80**	**<0.001**	**+0.48**	**+0.20**	**+0.77**
**1- 6 months** *(n=410)*	**+1.03**	**+0.15**	**+1.91**	**0.021**	**+0.16**	**+0.01**	**+0.30**
Over 6 months *(n=309)*	base						
**Not answered***(n=1)*	**-21.32**	**-33.26**	**-9.38**	**<0.001**	**-3.29**	**-5.27**	**-1.31**
**Primary carer of dog in 2020**[1]							
No *(n=2)*	+6.93	-1.22	+15.08	0.095			
Sole *(n=454)*	base						
Shared in household *(n=323)*	-0.36	-1.22	+0.51	0.418			
**Shared outside household** *(n=15)*	**-5.00**	**-8.08**	**-1.92**	**0.001**	**-0.77**	**-1.28**	**-0.25**
**Training method**[2]							
Mixed *(n=470)*	base						
Rewards only *(n=139)*	-0.13	-1.26	+0.99	0.816			
**Rewards, 1 aversive** *(n=162)*	**+1.21**	**+0.16**	**+2.25**	**0.024**	**+0.20**	**+0.03**	**+0.38**
Not answered *(n=23)*	-2.27	-4.73	+0.18	0.069			
**Aggression: Number of different problem behaviour types reported in 3 months prior to survey**[2]							
0 *(n=596)*	base						
1 *(n=138)*	-0.20	-1.29	+0.90	0.726			
2 *(n=37)*	+0.88	-1.08	+2.84	0.379			
**3 or 4** *(n=13)*	**-5.02**	**-8.31**	**-1.73**	**0.003**	**-0.77**	**-1.32**	**-0.22**
Not answered *(n=10)*	-2.69	-6.40	+1.01	0.154			
Constant:	***39.14***	***37.10***	***41.17***	***<0.001***			

Possible range of scores 10 – 50,
higher score represents increased closeness.
Month of birth and UK Region are random effects.
LRTest vs Linear model p = 0.996.
0.06% of unexplained variance due to random effects of region.

*z test for $H_0: \beta = 0$; bold indicate p < 0.05
[1]Data from 2020 survey.
[2]Data from survey when dogs 21 months old.
#Cohen's d_s in bold are over 0.5

Table 2: Mixed linear regression model for Perceived Costs (Costs) between Pandemic Puppy project owners and their 21-month old dogs acquired as puppies in July to December 2020 in the UK, measured by the PEC subscale of the Monash Dog-Owner Relationship Scale (n=794).

Variable	Category	Multi-variable β	95% Confidence Interval Lower	95% Confidence Interval Upper	p-value*	Effect size Cohen's d_s#	Effect size 95% CI Lower	Effect size 95% CI Upper
	Constant	***16.76***	***14.54***	***18.98***	***<0.001***			
**Respondent Age (years)**[1]								
	18 – 24 *(n=35)*	**-1.96**	**-3.90**	**-0.03**	**0.047**	-0.33	-0.70	+0.03
	25 – 34 *(n=153)*	base						
	35 – 44 *(n=155)*	-0.52	-1.69	+0.65	0.381			
	45 – 54 *(n=216)*	-0.75	-1.86	+0.37	0.188			
	55 – 64 *(n=144)*	+0.67	-0.61	+1.95	0.303			
	65 – 74 *(n=79)*	-0.17	-1.75	+1.40	0.829			
	75 + *(n=11)*	+2.40	-0.89	+5.68	0.153			
	No response *(n=1)*	-6.80	-17.18	+3.58	0.199			
**Respondent gender**[1]								
	Woman *(n=719)*	base						
	**Man** *(n=75)*	-1.00	-2.25	+0.25	0.118			
**Dog acquired as companionship for other people in household, not respondent** [1]								
	No *(n=708)*	base						
	**Yes** *(n=86)*	**+1.97**	**+0.79**	**+3.15**	**0.001**	+0.34	**+0.12**	**+0.55**
**C-19 pandemic influenced decision to acquire**[1]								
	No *(n=446)*	base						
	**Yes** *(n=321)*	**+1.50**	**+0.67**	**+2.32**	**<0.001**	**+0.26**	**+0.11**	**+0.40**
	**Not sure/no answer** *(n=27)*	**+4.48**	**+2.41**	**+6.55**	**<0.001**	**+0.77**	**+0.38**	**+1.16**
**Prior research**[1]								
	No *(n=15)*	-1.44	-4.18	+1.29	0.301			
	Yes *(n=462)*	base						
	**No, already experienced** *(n=315)*	**-1.61**	**-2.41**	**-0.80**	**<0.001**	**-0.27**	**-0.42**	**-0.13**
	**No answer** *(n=2)*	**+8.64**	**+1.30**	**+15.99**	**0.021**	**+1.47**	**+0.08**	**+2.86**
**Change in who involved in dog's care from <16 weeks to 21 months old**[2]								
	No *(n=206)*	base						
	**Yes** *(n=583)*	**-1.00**	**-1.87**	**-0.14**	**0.023**	**-0.17**	**-.033**	**-0.01**
	No answer *(n=5)*	+8.64	+1.30	+15.99	0.178			
**Owner takes dog with them if go out to work (dog 21 months old)**[2]								
	Don't go out or other adult takes dog *(n=354)*	base						
	Always *(n=13)*	-1.87	-4.79	+1.05	0.209			
	**At least half of workdays** *(n=22)*	**-3.33**	**-5.59**	**-1.06**	**0.004**	**-0.57**	**-1.00**	**-0.14**
	**Less than half of workdays** *(n=42)*	**-2.30**	**-3.99**	**-0.62**	**0.008**	**-0.39**	**-0.71**	**-0.07**
	Never *(n=359)*	-0.35	-1.18	+0.48	0.404			
	No answer *(n=4)*	+0.48	-4.66	+5.61	0.855			
**Training method**[2]								
	Mixed *(n=470)*	base						
	Rewards only *(n=139)*	-0.84	-1.84	+0.17	0.102			
	**Rewards, 1 aversive** *(n=162)*	**-0.99**	**-1.93**	**-0.06**	**0.038**	-0.17	-0.35	+0.01
	Not answered *(n=23)*	**-2.38**	**-4.56**	**-0.21**	**0.032**	-0.41	-0.83	+0.01
**Control problem: Number of different problem behaviours reported in 3 months prior to survey**[2]								
	0 *(n=145)*	Base						
	1 *(n=244)*	+1.05	-0.09	+2.20	0.071			
	2 *(n=274)*	+0.85	-0.26	+1.97	0.133			
	3 *(n=131)*	**+2.57**	**+1.25**	**+3.89**	**<0.001**	**+0.45**	**+0.20**	**+0.69**
	No answer *(n=20)*	+0.86	-1.77	+3.89	0.521			
**Aggression: Number of different problem behaviour types reported in 3 months prior to survey**[2]								

0 *(n=596)*	base						
1 *(n=138)*	-0.58	-1.56	+0.40	0.247			
2 *(n=37)*	+0.38	-1.44	+2.20	0.679			
**3 or 4** *(n=13)*	**+5.58**	**+2.57**	**+8.59**	**0.001**	**+0.95**	**+0.40**	**+1.51**
No answer *(n=10)*	+2.33	-1.10	+5.75	0.183			
**Fear/avoidance: Number of different problem behaviour types reported in 3 months prior to survey[2]**							
0 *(n=455)*	base						
1 *(n=198)*	-0.09	-0.97	+0.79	0.838			
2 *(n=93)*	**+1.58**	**+0.35**	**+2.81**	**0.012**	**+0.27**	**+0.05**	**+0.49**
3 or 4 *(n=34)*	**+2.18**	**+0.23**	**+4.12**	**0.028**	**+0.37**	**+0.02**	**+0.72**
No answer *(n=14)*	**+3.02**	**+0.12**	**+5.92**	**0.04**	+0.51	-0.02	+1.05
**Separation related behaviour[2]**							
No *(n=496)*	base						
Yes *(n=222)*	**+1.78**	**+0.94**	**+2.62**	**<0.001**	**+0.30**	**+0.15**	**+0.46**
No answer *(n=76)*	+0.21	-1.07	+1.48	0.749			
**Anticipated changes to household circumstances in next 3 months[2]**							
No *(n=692)*	base						
Easier to own dog *(n=43)*	**+1.69**	**+0.05**	**+3.32**	**0.043**	-0.29	-0.59	+0.02
Harder to own dog *(n=52)*	**+2.57**	**+1.08**	**+4.06**	**0.001**	-0.29	-0.57	0.00
No answer *(n=7)*	-4.34	-9.62	+0.94	0.108			
Constant	***16.76***	***14.54***	***18.98***	***<0.001***			

Possible range of scores 10 – 45,
higher score represents increased perceived costs.
Month of birth and UK Region are random effects.
LRTest vs Linear model p = 0.040.
2.55% of unexplained variance due to random effects of region.

[*]z test for H_0: $\beta = 0$; bold indicate $p < 0.05$
[1]Data from 2020 survey.
[2]Data from survey when dogs 21 months old.
[#]Cohen's d_s in bold CI does not include 0

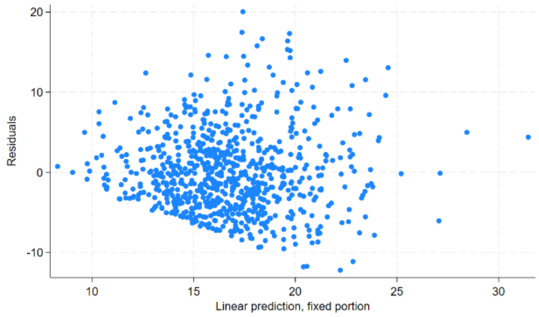

Figure 1: Plot of residuals versus predicted values for mixed linear regression model for Perceived Costs (Costs) between Pandemic Puppy project owners and their 21-month old dogs acquired as puppies in July to December 2020 in the UK, measured by the PEC subscale of the Monash Dog-Owner Relationship Scale (n=794).

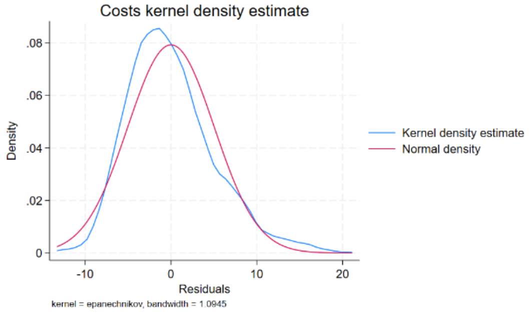

Figure 2: Normal curve and frequency of residual values for mixed linear regression model for Perceived Costs (Costs) between Pandemic Puppy project owners and their 21-month old dogs acquired as puppies in July to December 2020 in the UK, measured by the PEC subscale of the Monash Dog-Owner Relationship Scale (n=794).

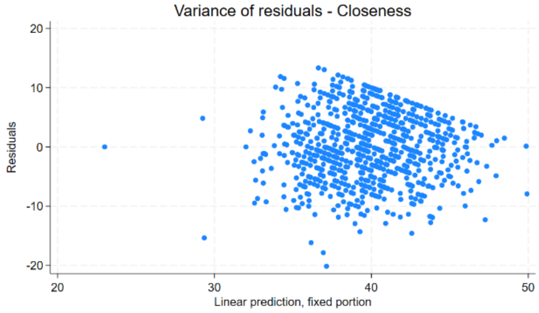

Figure 3: Plot of residuals versus predicted values for mixed linear regression model for Perceived Emotional Closeness (Closeness) between Pandemic Puppy project owners and their 21-month old dogs acquired as puppies in July to December 2020 in the UK, measured by the PEC subscale of the Monash Dog-Owner Relationship Scale (n=794).

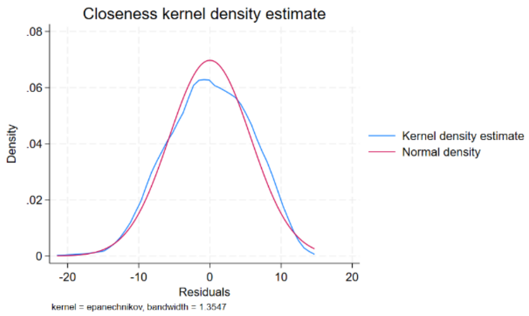

Figure 4: Normal curve and frequency of residual values for mixed linear regression model for Perceived Emotional Closeness (Closeness) between Pandemic Puppy project owners and their 21-month old dogs acquired as puppies in July to December 2020 in the UK, measured by the PEC subscale of the Monash Dog-Owner Relationship Scale (n=794).
