# Supplementary material for: Lasting lockdown love? Problem behaviour and pandemic and non-pandemic related risk factors influencing the owner-dog relationship in a UK cohort of dogs reaching early adulthood
Source: PLoS One. 2025 Feb 12;20(2):e0316166. doi: 10.1371/journal.pone.0316166 (PMC11819559; doi:10.1371/journal.pone.0316166)
Supplement: Attachment — Submitted filename: Review_PONE-D-24-29934.pdf [file pone.0316166.s005.pdf]

Review „Behaviour and other factors influencing the owner-dog bond as dogs reach early adulthood in the UK"

PONE-D-24-29934

This manuscript addresses a very important and interesting topic by aiming to identify risk factors associated with a weaker owner-dog bond in UK dogs acquired in 2020. The data provided is highly relevant to various areas of research in the human-animal bond and veterinary sciences.

The methodology and analyses are sound, carefully conducted and clearly described. However, I think that some aspects in the introduction and discussion could be better elaborated to improve the manuscript. I would also make a few changes to the helpful illustrations.

.

Title: After reading the manuscript, I felt that the title was too vague for the manuscript. It might be worthwhile to include "Pandemic dogs", "risk factors" instead of other factors that the title fits better to your actual investigations.

Introduction:

Line 47: Even though the first sentence makes it clear that emotional relationship in this manuscript is described by owner-dog bond, in my opinion the term is not consistently preserved. I would use either bond or relationship uniformly and consistently in the manuscript.

Line 58: I think it is important to clarify here that it is a matter of convenience euthanasia and would go into this aspect including moral challenges (also in veterinary practice) in more detail.

Line 63-69: As these are important factors for the analysis, these factors should not only be listed, but it would be helpful for the reader if the content would be unpacked. E.g. what is meant by lack of problem behaviour.

Line 72- 76: Same applies here. I think this information needs to be unpacked: What negative long-term effects on behaviour and health? I would provide precise examples and descriptions. Further, explain what is meant by "poor-welfare" source?

Line 84: "impact on dog welfare" This is an often-used term and should be clarified at the very beginning what is meant by this and in what way the dog welfare is impacted. Should be unpacked here and will be of help for the whole manuscript and your arguments.

Methods:

Risk factor variables and Table 1:

This table is a bit hard to follow and it might be a good idea to introduce some of them in a more detailed way in the introduction including your assumption in which direction the factors may impact owner-dog bond.

Line 179-183: Here, or in the introduction I would expect a short explanation of differences training methods. This would be further helpful for the discussion.

Results:
Line: 291: “%” is missing

Figure 2: In general, I really like this figure and it provides a good overview of your results. For publication, a better resolution of the figure is needed. The resolution is currently very poor and hard to read.
Few suggestions:
Line 336-343: As this is a really long heading for a table, you can think about to replaces info (Line 336-343) in footnotes

“No change in care provision” and “Change in care provision” reads a bit irritating and it is not very clear what is meant by this while exploring your figure. Might be better to choose a more precise description that reflects this aspect.

Figure 3: In general, I really like this figure and it provides a good overview of your results. For publication, a better resolution of the figure is needed. The resolution is currently very poor and hard to read.
Line 376-382: As this is a really long heading for a table, you can think about to replaces info (Line 336-343) in footnotes

“<6m to acquire” → I would include “decision” here

Line 399-401: Again, an explanation for reader who are not familiar with certain training techniques, would be of help.

Discussion:
Line 419: “impact” instead of “harm”
Line 421: Again, what is meant by dog welfare? See comment for introduction.
Line 428-434: Methods section?
Line 434-436: Should go in the limitation section

Line 441: I would provide more details for each aspect.
Line 445-448: As this is an ongoing debate, I would expect a bit more reflection on euthanasia based on aggressive behavior.

Line 471-476: I think a huge problem is that most of the people are not aware about the time, money they have to spend and too little responsibility is taken by the owners.
Line 501: Not only ability, but also awareness of their responsibilities.
Line 507: Some countries have already introduced a certificate of competence, so that a dog may only be bought after a two-day course. This is also a ‘hurdle’ and can protect against impulse buying.
Line 512-517: Further explanation: The desire to get a dog may have existed for a long time and it was during this phase (with the hype) that the purchase was made. I think that should be taken into account. The decision to buy is different from the phase before that: Have you wanted a dog for a long time?

Line 523-525: Could also be the feeling that you are doing justice to both: The dog and the work

Line 527-536: A bit weak and lacking argumentation for the discussed results.

I wish the authors good luck with the publication and look forward to the final publication of the highly relevant data.
